# Environmental and Occupational Respiratory Disease – 1030: Role of fungi (molds) in allergic airway disease – an analysis in South Indian Otolaryngology Center

**DOI:** 10.1186/1939-4551-6-S1-P29

**Published:** 2013-04-23

**Authors:** Vattikonda Sathavahana Chowdary

**Affiliations:** 1 Head & Neck Surgery, Allergy Clinic., Apollo Hospitals, Jubilee Hills, Hyderabad, India

## Background

To determine the incidence of Fungal sensitivity and to identify the types of fungi responsible for causing Respiratory Allergies in South Indian population.

## Methods

A study was done on the data related to 570 patients who had visited the allergy clinic in the Otolaryngology Department from January 2000 to September 2009. Clinical history related to Allergy was taken and clinical examination was done. The investigations done included Total Serum IgE levels, Peripheral Oesinophil percentage, Skin tests & Fungal culture. Based on the clinical findings, patients were categorized into three groups viz.1. Allergic Rhinitis (AR), 2. Allergic Rhinitis with Asthma (ARA), 3. Allergic Fungal Sinusitis (AFS). Based on the skin tests, patients were classified into three groups viz. 1. Positive towards fungus, 2. Positive towards allergens other than fungus (HDM, pollen, insects, food, etc), 3. Positive towards other allergens and fungus. Based on the skin tests, individuals sensitized to specific type of fungi were identified.

## Results

The results for sensitivity on skin tests were as follows: Fungus alone: 29.9%; Fungus + other allergens: 14.7%; Other allergens: 55.3%. Whereas, Overall sensitivity for fungus: 29.9% + 14.7% = 44.7%; Positivity for fungus alone was highest in AR group (47.9%); Positivity for fungus + other allergens was highest in AR group (63.8%). In all three clinical groups, the highest sensitivity for fungus that was identified was for *Aspergillus fumigatus* and *Aspergillus flavus*. In case of Allergic Fungal Sinusitis with Nasal Polyposis, the most common fungus that was identified on fungal culture was *Aspergillus fumigatus* (55%) followed by *Aspergillus flavus* (40%). Skin tests conducted by Agashe *et al* (Bangalore, South India) were found to be positive for *Helminthosporium*, *Alternaria nigrospora* and *Cladosporium* (Agashe SN *et al*, 1983).

## Conclusions

Diagnosis and Immunotherapy of allergy to fungi require well characterized or standardized extracts that contain the relevant allergens of the appropriate fungus. More such studies from India and other countries may help in the better understanding of this condition which can lead to proper diagnosis and management.

